# An Approach to Identify Individual Functional Single Nucleotide Polymorphisms and Isoform MicroRNAs

**DOI:** 10.1155/2019/6193673

**Published:** 2019-07-30

**Authors:** Ying Wang, Jidong Ru, Tao Jin, Ming Sun, Lizhu Jia, Guijiang Sun

**Affiliations:** ^1^College of Computer and Control Engineering, Qiqihar University, No. 42, Wenhua Street, Qiqihar, Heilongjiang 161006, China; ^2^College of Light Industry and Textile, Qiqihar University, No. 42, Wenhua Street, Qiqihar, Heilongjiang 161006, China; ^3^Network Information Center, Qiqihar University, No. 42, Wenhua Street, Qiqihar, Heilongjiang 161006, China

## Abstract

MicroRNAs (miRNAs) and single nucleotide polymorphisms (SNPs) play important roles in disease risk and development, especially cancer. Importantly, when SNPs are located in pre-miRNAs, they affect their splicing mechanism and change the function of miRNAs. To improve disease risk assessment, we propose an approach and developed a software tool, IsomiR_Find, to identify disease/phenotype-related SNPs and isomiRs in individuals. Our approach is based on the individual's samples, with SNP information extracted from the 1000 Genomes Project. SNPs were mapped to pre-miRNAs based on whole-genome coordinates and then SNP-pre-miRNA sequences were constructed. Moreover, we developed matpred2, a software tool to identify the four splicing sites of mature miRNAs. Using matpred2, we identified isomiRs and then verified them by searching within individual miRNA sequencing data. Our approach yielded biomarkers for biological experiments, mined functions of miRNAs and SNPs, improved disease risk assessment, and provided a way to achieve individualized precision medicine.

## 1. Introduction

MicroRNAs (miRNAs) are 18-24 nucleotides (nts) long, sing-stranded, noncoding RNAs. The biogenesis of miRNAs generally follows a canonical process: primary miRNAs (pri-miRNAs) are cleaved by the RNase III Drosha enzyme, generating precursor miRNAs (pre-miRNAs). These pre-miRNAs are transported to the cytoplasm and further cleaved into 22 nts long miRNA:miRNA*∗* duplexes under the action of the RNase III Dicer enzyme [1]. Only miRNAs are loaded into the RNA-induced silencing complex for mRNA transcript target recognition [2]. Reliance of miRNA biogenesis on sequence can determine the typical secondary structure and thermodynamics for correct processing, and sequence variation around processing sites, mature miRNAs, and flanking regions of pre-miRNAs lead to changes in base-paring, structure, stability, and thermodynamics. Therefore, a single nucleotide polymorphism (SNP) in miRNAs may influence processing [3, 4] and disrupt expression, biosynthesis, or activity of miRNAs, altering miRNA biogenesis and function [5, 6]. On the other hand, the recent advent of next-generation sequencing showed that mature miRNAs present some sequence variants, or isoforms, with corresponding “reference” mature sequences that generate multiple variants named isoform miRNAs (isomiRs) [7, 8]. Recent studies showed that SNPs may affect the miRNA maturation process [9], and isomiR generation is mainly attributed to imprecise cleavage by Drosha and Dicer, RNA editing, and SNPs, taking miR-934-T/G for example, variant occurs at Drosha sit, it leads to 5p product reduce, and isomiRs with longer sequence than 5p increased, most dramatic change happens in the 3p product, the effect of SNP in mutant samples gives rise to isomiRs with mutant type in the Drosha sit, and variant produces more isomiRs than canonical mature miRNA resulting from wild type [10].

Given that impaired miRNA processing can lead to substantial decrease in miRNA and increase in isomiRs expression levels, and isomiRs differentially regulate the targeted mRNA transcripts, complex phenotypes and diseases may result [8]. SNPs in miRNA can thus have significant phenotypic consequences and cause various diseases. Because SNPs are inherited genetic variations, they can be detected by high-throughput technology, and, therefore, SNPs and isomiRs are alternative or complementary markers to tissue-based biomarkers. Identification of SNPs and isomiRs is of great significance to explore the functions of SNPs and miRNAs, revealing different human phenotypes and disease risk.

In recent years, owing to the important roles of SNP-related miRNA (SNP-miRNA) in disease risk and development, several databases and software tools such as MirSNP [11], PolymiRTS [12], SubmiRine [13], MicroSNiPer [14], Mirsnpscore [15], and mrSNP [16] were developed. However, these databases mainly focus on predicting the effects of SNPs on miRNAs targets. For the study SNP and isomiR function, a series of isomiR-related databases and software tools were developed. IsomiRex can identify miRNA, isomiRs, and differential expression based on next-generation sequencing data [17]. miR-isomiRExp analyzes the expression levels of miRNAs/isomiRs and track miRNA/isomiR processing mechanisms to uncover functional characteristics of these molecules [18]. The isomiR databank collected 308,919 isomiRs associated with 4,706 mature miRNAs, revealing the function of these isomiRs [19]. miRNASNP is a SNP-related miRNA database based on miRBase and dbSNP databases and contains 2257 SNPs located in 1596 pre-miRNA loci and flanking regions [20]. MSDD captured experimentally validated relationships between miRNAs, SNPs, genes, and diseases and contains 182 human miRNAs, 197 SNPs, 153 genes, and 525 interactions between them [20]. In addition, MiRVaS provides positions of mutations in miRNA (seed, maturation, stem, ring, hairpin arm, and flanking region) and predicts the changes that these variations will cause in miRNA structure [4]. MiRvar studied the effects of SNPs on the miRNA maturation mechanism, extracted 106 SNPs located in 85 miRNAs, and identified the typical miRNA and related isomiRs by analyzing the allele frequencies of 23 SNPs, suggesting that these SNPs have specific functions [21].

The study of SNP-miRNA focuses on several aspects: prediction of the effects of SNPs on miRNA targets, SNP-miRNA database development, the relationship of SNP-miRNA with disease or expression change, and identification of SNP-miRNA-related isomiRs using next-generation sequencing. The mechanism of mature miRNA processing should be further studied. Moreover, alternate splicing caused by variants was predicted by computational methods, and it was verified that up to 94 % of variants causing alternate splicing were correctly classified [22]. Because the mature sequence and processing mechanism can be predicted based on the biological characters of miRNAs using machine learning methods, several software tools were employed to identify the mature miRNAs (processing sites) from a given pre-miRNA, such as miRdup [23], miRmat [24], MatureByes [25], mirExplore [26], MaturePred [27], MiRPara [28], miRRim2 [29], Microprocessor SVM [30], and MiRduplexSVM [31].

In our previous study, we developed software to predict mature miRNAs [32, 33] from a novel pre-miRNA, and the 1000 Genomes Project contains the genomes of 2504 individuals from 26 populations. It includes more than 88 million variants, 3.6 million short insertions/deletions, and 60,000 structural variants, and the distribution of genetic variations across the global sample plays important roles in disease [34]. However, few studies have focused on the identification of SNPs and isomiRs from individual samples. Considering the urgent needs of individualized precision medicine, in this study we provide an approach for identifying individual functional SNPs and the corresponding isomiRs, SNPs, and related pre-miRNAs from individual samples.

## 2. Materials and Methods

### 2.1. Data

Pre-miRNAs data (miRNA ID, Chromosome, Start position coordinate, End position coordinate, and strand) from 1881 human pre-miRNAs were downloaded from miRBase (release V20). SNPs data (CHROM, POS, REF, ALT, AF, and GT) of individual samples, including 23 Chromosome data, were obtained from the 1000 Genomes Project (ftp://ftp.1000genomes.ebi.ac.uk/vol1/ftp/release/20130502/). The human GRCh37 reference sequence (human_g1k_v37.dict, human_g1k_v37.fasta, and human_g1k_v37.fasta.fai) was downloaded from ftp://ftp.1000genomes.ebi.ac.uk/vol1/ftp/technical/reference/. miRNA sequencing data from individual samples in the 1000 Genomes Project were obtained from https://www.ebi.ac.uk/arrayexpress/experiments/E-GEUV-2/samples/.

### 2.2. SNPs Extraction from Individual Samples

The 1000 Genome Project contains the characteristics and distribution of common and rare variations from individuals of 26 populations. We download the VCF file, a generic format for storing DNA polymorphism data such as SNPs, insertions, deletions, and structural variants, together with rich annotations of individual samples from the 1000 Genome Project. The attributes of SNP information can be listed as follows:

(1) Attrsnp=CHROM,QUAL,SNPID,FILTER,Alter,Qual,Filter,INFO,STRAND

where CHROM corresponds to chromosome, POS, a 1-based position of the start of the variant, ID, a unique identifier of the variant, REF, the reference allele, ALT, a comma separated list of alternate nonreference alleles, QUAL, a quality score, FILTER, site filtering information, and INFO, a semicolon separated list of additional user extensible annotation.

The software GenomeAnalysisTK.jar was used to extract chromosome data and the VCF file of the 23 chromosomes from each sample. During this process, the files including human_g1k_v37.dict, human_g1k_v37.fasta, and human_g1k_v37.fasta.fai were used for SNP search within the human reference sequence. All 23 chromosomes from each example were integrated into one file presenting all SNPs and SNP-pre-miRNAs.

### 2.3. Construction of SNP-Pre-miRNA Sequences

To construct SNP-pre-miRNA sequences, SNPs must be mapped to the pre-miRNAs based on whole-genome coordinates. The coordinates of pre-miRNA can be extracted from miRNA information, and thus we downloaded pre-miRNA data from miRBase (V20). The attributes of miRNAs can be described as follows:

(2) Attrmir=miRNA  name,Genome  reference  sequence  alliance,chromosome,Start  position  coordinates,End  position  coordinates,Strand

We extracted pre-miRNA and SNP data based on start and end position coordinates of pre-miRNAs and the start position of SNP.

SNPs and miRNAs in the human genome sequence may be located in the plus or trans-strand and, therefore, the coordinates of SNPs and pre-miRNAs must be converted. The SNPs positions of each pre-miRNA in the whole-genome coordinates were calculated. Next, in the pre-miRNA sequences, the SNPs position in the pre-miRNA was searched from front to back, and the trans-strand was searched from back to front to find the corresponding nucleotides.

**Figure 1:**
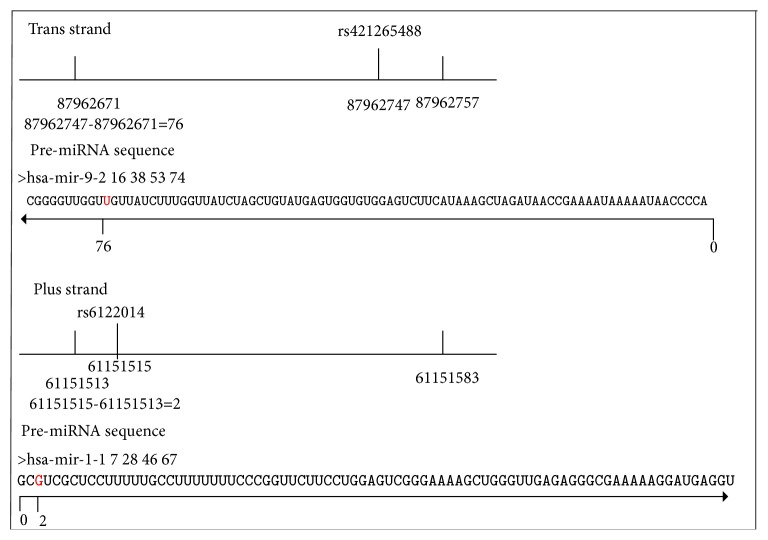
Calculation of SNPs position based on the plus- and trans-strands.

Taking hsa-mir-9-2 and hsa-mir-1-1 as examples, SNP rs41265488 is located in the trans-strand, and we thus calculated the number of nucleotides between pre-miRNA and SNP as 76 and then located the SNP position in the pre-miRNA from back to front, and the number of spacer nucleotides is 76. SNP rs6122014 in has-mir-1-1 is located in the plus strand, and the number of nucleotides between pre-miRNA and SNP is 2, and thus we located the SNP position in the pre-miRNA from front to back, and the number of spacer nucleotides is 2. Calculation of SNPs position based on plus- and trans-strands is shown in Figure 1.

Each pre-miRNA and one or more SNPs can then be mapped based on position coordinates. Because a pre-miRNA may include one or more SNPs, each SNP was used to substitute canonical nucleotides in the pre-miRNA. The combination method was used to construct the SNP-pre-miRNAs of each pre-miRNA:

(3) $$\begin{array}{c} N_{i}=C_{n}^{1}+C_{n}^{2}+\ldots +C_{n}^{k}+\ldots +C_{n}^{n} \end{array}$$

where *N*_*i*_ is the number of SNP-pre-miRNA of the i^th^ pre-miRNA and n is the number of SNPs of the i^th^ pre-miRNA.

Using this method, all possible SNP-pre-miRNA sequences can be obtained without losing important information.

### 2.4. IsomiR Identification in SNP-Pre-miRNAs

In our previous study, we developed matpred [33] to identify mature miRNAs from pre-miRNAs using biological characteristics of the 5′ arm start sites. As is known, isomiRs are always generated from 3′ arm heterogeneity [8, 10]. Therefore, we developed an extended version of matpred, named matpred2, a software tool capable of identifying mature miRNA based on all processing site characteristics. The underlying reasoning of matpred2 is to consider the four processing sites as identified objects. Firstly, we extracted the training dataset based on the four processing sites. Next, the biological features from each site were extracted, and at last the classifier model was trained. With the exception of dataset extraction training, the other steps of the training model, including feature extraction, feature selection, and model training, are those of matpred.

We extracted 115 features which represent the characteristic of mature miRNA: all features were shown in Table 1:

Minimum free energy feature including MFE of miRNA:miRNA*∗* duplex (MFE1), flank region from 9nt on the left of 5′arm Drosha sit to Dicer sit (9nt-MFE), flank region from 5nt on the left of 5′arm Drosha sit to Dicer sit (5nt-MFE), flank region from3nt on the left of 5′arm Drosha sit to Dicer sit (3nt-MFE), and flank region from 3nt on the right of 5′arm Drosha sit to Dicer sit (+3nt-MFE).

Structural specificity feature is the nucleotide and structural composition at each site. Taken M and N represent paired nucleotide and no-paired nucleotide, then structural specificity feature is one of the following combinations: AM, CM, GM, UM, AN, CN, GN, UN, -N. We extracted 50, 18, 6 structural specificity feature of miRNA:miRNA*∗* duplex, flank region of 9nt on the left of 5′arm Drosha sit (Left 9nt flank region) and flank region of 3nt on the right of 5′arm Dicer sit(3nt flank region).

Paired nucleotide:Each sit nucleotide pairing information of miRNA:miRNA*∗* duplex. The specific characteristics are as follows: AA, AC, AG, AU, CA, CC, CG, CU, GA, GC, GG, GU, UA, UC, UG, UU, A-, C-, G-, U-, -A, –C, –G, –U.

In addition, we extracted the number of “-” in sequence. In particular, from 3nt to 8nt on the right of Drosha, from 9nt to 12nt on the right of Drosha, and from 2nt on the left of Drosha to 2nt on the right of Drosha were extracted. And the distance from 5′arm Drosha sit to terminal loop was adopted.

Owing to the models used for each site, matpred2 lacks the low recognition accuracy problem caused by the uncertainty in the 5′ and 3′ ends of the mature miRNA length and migration distance that affects the recognition of mature miRNA in double-stranded sequence. In addition, based on the biological feature differences between Drosha and Dicer sites, the two sites were separated in the prediction model to make the classifier more representative.

For each processing site of SNP-pre-miRNA, matpred identifies five mature miRNA candidates. To improve the accuracy of our prediction results and remove false positive data, the five candidates are all considered as canonical miRNA. In consequence, miRNAs that differed from the candidates were identified as isomiRs. Related SNPs were identified as functional SNPs.

### 2.5. Software Development

To make our approach convenient for application, we provide IsomiR_Find, which is designed to identify individual SNPs and SNP-pre-miRNAs. All dicer sites of mature miRNAs were identified using matpred2 [32]. Moreover, mature miRNA candidates were predicted to provide the normal miRNA and isomiRs, and experimentally validated isomiRs were screened based on miRNA sequencing data.

## 3. Results and Discussion

### 3.1. Approach Design and Implementation

**Figure 2:**
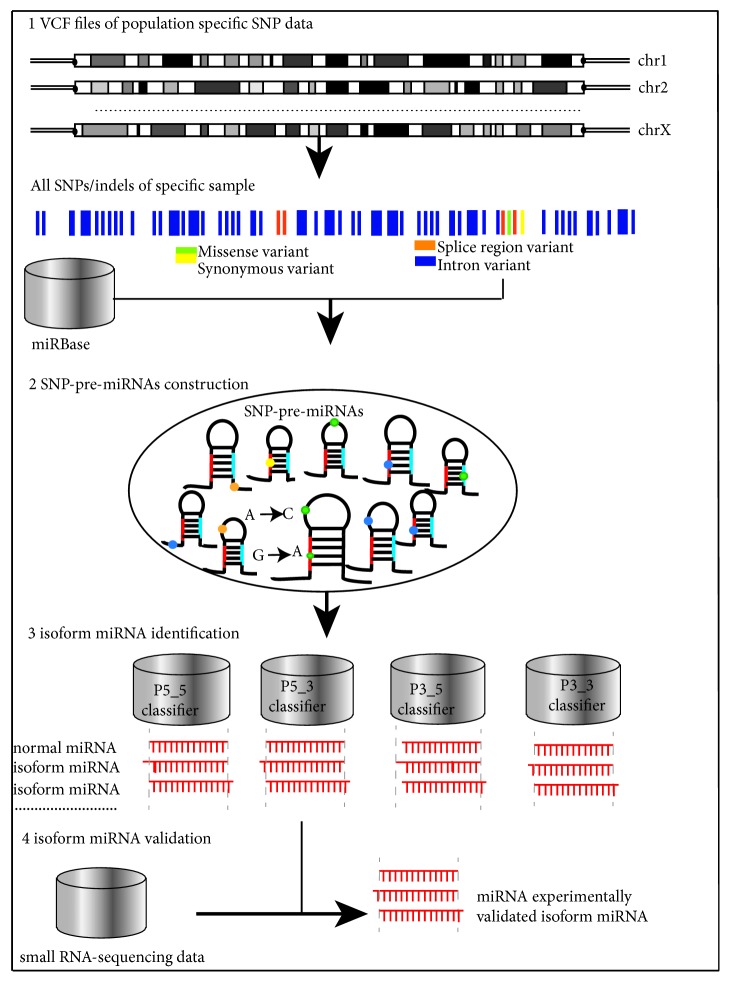
Computational pipeline to identify individual functional SNPs and corresponding isomiRs.

A computational pipeline was established to identify individual functional SNPs and corresponding isomiRs as shown in Figure 2.

Firstly, we extracted the SNPs of each chromosome from the VCF files. Secondly, we combined all the SNPs of each chromosome by alignment of their positions with miRBase, obtained the SNPs and related pre-miRNAs of each sample, and then constructed the SNP-pre-miRNA sequences of this sample. Thirdly, we identified the four cleavage sites of these SNP-pre-miRNAs using matpred2, and then the normal miRNA and isomiR candidates were extracted. Finally, the individual miRNA sequencing data were used to validate and mine the isomiRs.

### 3.2. SNP Extraction from Individual Samples

To test our approach, samples from a British population in England and Scotland (GBR) were selected. GBR data from the 1000 Genome Project includes DNA polymorphism and miRNA sequencing data. DNA polymorphism data of each sample were obtained based on karyotype, and, using our approach, we extracted the SNPs of each chromosome and integrated the SNPs of all chromosomes.

**Figure 3:**
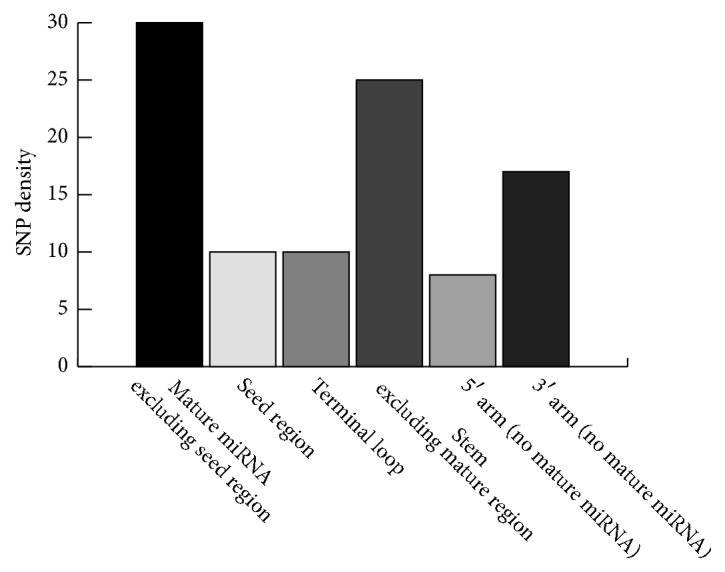
SNP location in pre-miRNAs from mapped data of sample HG00096 from the 1000 Genomes Project.

Taking one sample from GBR, HG00096, as an example, our approach mapped SNPs on genomic locations of pre-miRNAs, and 96 SNPs were identified in 90 pre-miRNAs (Supplementary Table 1). SNP location on pre-miRNAs from the mapped data of sample HG00096 is shown in Figure 3.

A total of 30 SNPs were identified in the mature miRNA region, 10 in the seed regions, 10 in the terminal loop, and 25 in the stem region. The results show that the mature miRNA region of pre-miRNA has the highest, and the terminal loop the lowest, SNP density.

### 3.3. Construction of SNP-Pre-miRNAs

SNPs were mapped to pre-miRNAs based on their positions in the genome. For example, if miRNA end and start positions are 33484789 and 33484781, respectively, and the position of the SNP rs114964240 is 33484783, then the SNP is located in has-mir-187.

**Figure 4:**
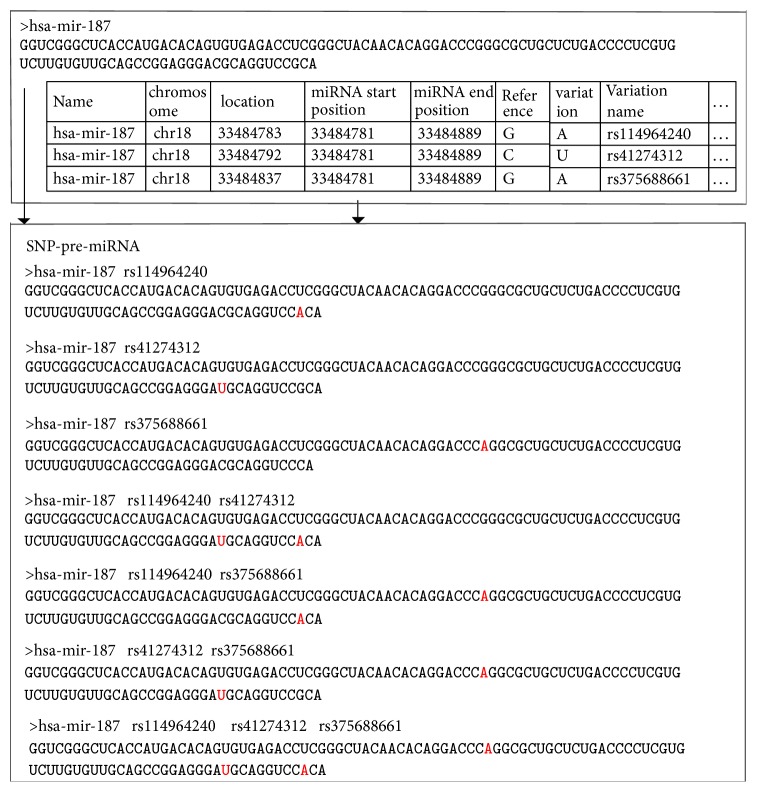
Construction of the SNP-pre-miRNA sequences of HG0096.

The location of SNPs in pre-miRNA is calculated based on coordinates and the trans/plus strand. For example, rs114964240 is located in the trans-strand, and the distance of re114964240 from the miRNA start position of has-mir-187 is 2. The SNP position in the pre-miRNA is then calculated from back to front of the pre-miRNA sequence, and, therefore, in this SNP, A substitutes G. We then constructed the SNP-pre-miRNA sequences of HG0096 as shown in Figure 4.

Taking has-mir-187 as an example, three SNPs were mapped into has-mir-187 based on the combination method, and we constructed seven SNP-pre-miRNA sequences. The SNP-pre-miRNA sequences of HG00096 are shown in Supplementary Table 2.

### 3.4. IsomiR Identification

**Figure 5:**
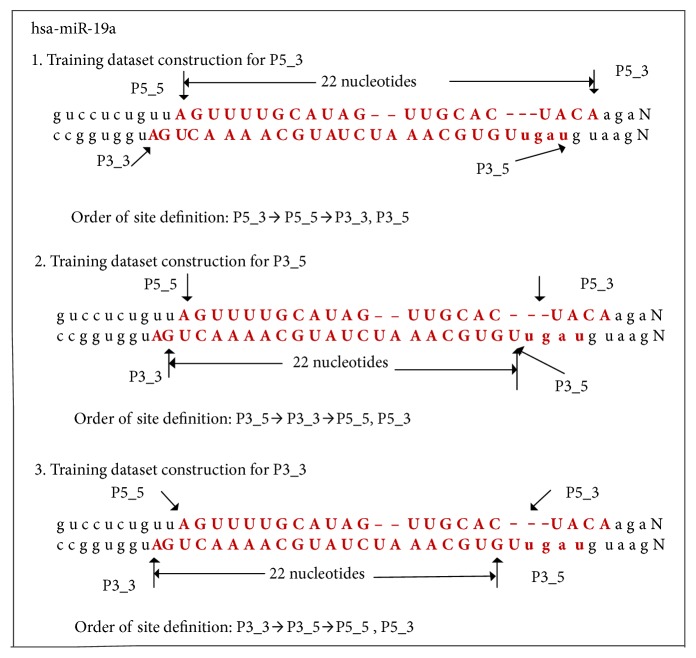
Construction of training datasets for the P5_3, P3_5, and P3_3 classifiers using hsa-miR-19a as an example.

Matpred2 was trained to identify the four sites of mature miRNAs of pre-miRNA. The four classifiers were trained as follows: training dataset construction, feature set extraction, feature set selection, and classifier training. We defined the start and end sites of the 5′ arm of pre-miRNAs as P5_5 and P5_3, respectively, and the start and end sites of the 3′ arm of pre-miRNAs as P3_5 and P3_3, respectively. The main difference between the training of classifiers P5_3, P3_5, and P3_3 lies in the construction of the training data sets: the P3_5 classifier used the 5_for_train dataset, and the P5_3 and P3_3 classifiers used the 3_for_train dataset. Taking hsa-mir-19a as an example, the construction of the training sets of the P5_3, P3_5, and P3_3 classifiers is shown in Figure 5.

To construct the training dataset of the P5_3 classifier, the 22^nd^ nucleotide in front of P5_3 was defined as P5_5, and the sequence between P5_5 and P5_3 was shifted to the left of the 3′ end. Two corresponding sites of the 2 nt sequence are P3_3 and P3_5. The sequence between P5_5 and P5_3 is positive example data, whereas all sequences offset by 1 nt distance are negative example data. Similarly, the training datasets of P3_5 and P3_3 were constructed. Feature set extraction, feature set selection, and classifier training were performed as described for matpred [33]. Specifically, for P5_5, P5_3, P3_5, and P3_3, the position deviation predicted accuracies of the first candidate are 79%, 71%, 66%, and 90%, respectively within 5nt distances.

To investigate the potential functional effects of SNPs in pre-miRNAs, we used matpred2 to identify mature miRNAs of SNP-pre-miRNAs of HG00096 (Supplementary Table 3). We predicted 101 pre-miRNAs having 115 SNPs in a different guide strand with the incorporation of variations in its sequence. The pre-miRNAs, SNPs, and potential functional effects are summarized in Table 2. All SNPs within pre-miRNAs could have a potential impact in biogenesis and function.

For the four dicer sites, we identified 11 SNPs within 7 pre-miRNAs, 39 SNPs within 47 pre-miRNAs, 11 SNPs within 12 pre-miRNAs, and 44 SNPs within 45 pre-miRNAs with the potential function to alter P5_5, P5_3, P3_5, and P3_3 sites, respectively.

### 3.5. IsomiR Validation

Although SNPs in pre-miRNAs were predicted to alter miRNA splicing sites and generate isomiRs, to validate these effects in experiments, we downloaded the miRNA sequencing data of HG00096 and searched isomiR sequences with reads from the miRNA sequencing data. We identified isomiRs using matpred2 and validated them in miRNA sequencing data as shown in Table 3.

Mature miRNAs were identified in all SNP-pre-miRNAs using the four classifiers. For P5_5, we identified isomiRs of hsa-mir2173h, and then we searched these isomiRs in the miRNA sequencing data of HG00096. We found four isomiRs with the following sequences: CCTGGGAGGTCAAGGCTGTAGT, GCCTGGGAGGTCAAGGCTGTAG, ATTGCTTGAGCCTGGGAGGTCA, and TTGAGCCTGGGAGGTCAAGGCT. The corresponding number of occurrences was 1, 1, 11, and 24 reads, respectively, in the miRNA sequencing data.

For HG00096, we searched 36 isomiRs within 11 pre-miRNAs in the miRNA sequencing data.

### 3.6. Software Implementation

IsomiR_Find was developed in perl and JavaScript. To use IsomiR_Find, the files human_g1k_v37.dict, human_g1k_v37.fasta, and human_g1k_v37.fasta.fai were needed to extract individual SNPs, as well as GenomeAnalysisTK.jar, libsvm-3.20, and ViennaRNA-1.8.5. The user selects the sample for study from the 1000 Genomes Project database, and IsomiR_Find identifies its SNPs and associated isomiRs. IsomiR_Find is freely available at https://github.com/wangying0128/IsomiR_Find. For the convenience of users, we provide a virtual machine image of the installed IsomiR_Find tool to use IsomiR_Find in windows system, it can be downloaded from https://pan.baidu.com/s/1YCQQYy-RMT0O5hJY8UvYOA.”

IsomiR_Find is a freely accessible tool for identify candidate SNPs and related isoform miRNAs in individuals from DNA sequencing data; especially, it can also predict isomiRs for novel miRNA sequences with novel SNPs. Owing to MatPred2 including four models for predicting four dicer sits of 5′ and 3′ arm, our tool can predict the isomiR from both the arms. Because MatPred2 is trained based on conservation features of pre-miRNA, it can be used to identify mature miRNAs of novel pre-miRNAs. Therefore, isomiR can predict various types isomiR such as 5′addition, 5′trimming, 3′addition, 3′ trimming, 3′ nontemplate addition, and 5′ nontemplate addition no matter with seed SNP or tail SNP.

## 4. Conclusions

MiRNAs and SNPs play important roles in diseases. miRNA function is closely related to its generation mechanism, and SNPs located in the pre-miRNA may affect its function. To improve personal disease risk assessment, we developed an approach to identify heterogeneous isomiRs generated by an individual's SNPs affecting the pre-miRNA maturation mechanism.

For this approach, we developed a freely accessible software tool, IsomiR_Find, to identify disease-causing candidate SNPs and associated miRNAs in individuals, and screen candidate SNPs and related isomiRs. The presented algorithm is, to our knowledge, the first approach that aims at isomiR identification in individuals. It will provide a deeper understanding of transcriptome mutation, cell mechanism discovery, and SNP functional exploration in the human genome. Furthermore, identification of relevant isomiRs can provide a valuable reference for biological experiments, and it can be used to investigate the relationship between SNPs in individuals and diseases. Our approach and the software IsomiR_Find are freely accessible for identification of individual SNP- pre-microRNAs and related isomiRs. The software facilitates the identification of candidate disease-causing SNPs and associated miRNAs in individuals and the screening of candidate SNPs and related isomiRs for experimental validation.

Our approach is applied to the 1000 genomes project which detect most variants with frequencies as low as 1% for 2,504 samples. Our approach mined single nucleotide variation (SNV) functions from the perspective of SNV affecting the miRNA maturation mechanism, providing a new idea for the study of SNV functions in noncoding regions. Because our approach is based on DNA sequence, in clinical applications it can be used to analyze the effects of each SNV on miRNA processing in the individual's DNA sequence with one drop of blood and then predict the function of SNV on phenotype and disease. In addition, our approach solves the technical bottlenecks arising from a small number of heterogeneous miRNAs, low SNV allele frequency, and undesirable miRNA sequencing samples and is expected to make breakthrough discoveries in the function of heterogeneous miRNAs and SNVs in diseases and phenotypes.

Our tool focus on identifying isomiRs which derived from SNP effects on miRNAs mature mechanism. In addition, the origins of isomiRs also include imprecise cleavage of Drosha and Dicer, RNA editing, 3′ addition events, and TRBP regulation; it should be studied in further work.

As we known, 5′isomiR will have a large influence on target specificity as it has seed sequences, so an effective tool that provides a target can significantly understand their molecular mechanism; we shall make efforts in our future work to provide a tool to predict the target gene for isomiRs.

## Figures and Tables

**Table 1:** The feature set of mature miRNA identification.

category	Mature miRNA(number)	MiRNA:miRNA*∗*duplex(number)	Pre-miRNA	Pri-miRNA
			(number)	(number)
length			Distance to terminal loop(1)	

minimum free energy		MFE1(1)		9nt-MFE; 5nt-MFE; 3nt-MFE; +3nt-MFE; (4)

Structural specificity		(50)		Left 9nt flank region(18); right 3nt flank region(6)

Paired nucleotide		Paired nucleotide(25)		

Number of “-”	3-8nt; 9-12nt; -2-2nt; (3)			

Paired nucleotide	First nucleotide type(1); First nucleotide paired(1); Single nucleotide frequency(5)			

**Table 2:** Pre-miRNAs, SNPs, and potential functional effects of HG00096.

Category	pre-miRNA	SNP	Effect of SNPs
Number	7	11	Altered P5_5
Number	39	47	Altered P5_3
Number	11	12	Altered P3_5
Number	44	45	Altered P3_3
Total	101	115	

SNP, single nucleotide polymorphism; miRNA, microRNA.

**Table 3:** Identification of isomiRs using matpred2 and validation using miRNA sequencing data.

Sits	Name	Mature miRNA	Number	Mature miRNA	Number	Mature miRNA	Number
P5_5	>hsa-mir-1273h	CCTGGGAGGTCAAGGCTGTAGT	1	GCCTGGGAGGTCAAGGCTGTAG	1	ATTGCTTGAGCCTGGGAGGTCA	11
P5_5	>hsa-mir-1273h	TTGAGCCTGGGAGGTCAAGGCT	24				
P5_3	>hsa-mir-1273d	GAGGTTGAGGCTGCAGTGAGCC	10				
P5_3	>hsa-mir-1273d	GAGGTTGAGGCTGCAGTGAGCC	10	TGAGGTTGAGGCTGCAGTGAGC	1		
P5_3	>hsa-mir-564	TCAGCAGGCAACATGGCCGAGA	1	TGTCAGCAGGCAACATGGCCGA	1	GTCAGCAGGCAACATGGCCGAG	1
P5_3	>hsa-mir-564	GTGTCAGCAGGCAACATGGCCG	1				
P5_3	>hsa-mir-663a	TCCCAGGCGGGGCGCCGCGGGA	2	TCCGGCGTCCCAGGCGGGGCGC	3		
P5_3	>hsa-mir-635	TGAAACAATGTCCATTAGGCTT	1	GAAACAATGTCCATTAGGCTTT	1	ACAATGTCCATTAGGCTTTGTT	1
P5_3	>hsa-mir-635	AACAATGTCCATTAGGCTTTGT	1	CTGAAACAATGTCCATTAGGCT	1		
P5_3	>hsa-mir-1254-2	TGGAAGCTGGAGCCTGCAGTGA	1	TGAGCCTGGAAGCTGGAGCCTG	1	GCCTGGAAGCTGGAGCCTGCAG	1
P5_3	>hsa-mir-1273h	TGAGCCTGGGAGGTCAAGGCTG	5	TTGAGCCTGGGAGGTCAAGGCT	4	TGCTTGAGCCTGGGAGGTCAAG	4
P5_3	>hsa-mir-1273h	TTGCTTGAGCCTGGGAGGTCAA	2				
P3_5	>hsa-mir-320e	GAAAAGCTGGGTTGAGAAGGT	1	AAAAGCTGGGTTGAGAAGGT	1	GGAAAAGCTGGGTTGAGAAGGT	1
P3_3	>hsa-mir-3615	CTCTCTCGGCTCCTCGCGGCTC	1	GGCTCCTCGCGGCTCGCGGCGG	1	CGGCTCCTCGCGGCTCGCGGCG	1
P3_3	>hsa-mir-3615	TCGGCTCCTCGCGGCTCGCGGC	1				
P3_3	>hsa-mir-1303	TAGAGACGGGGTCTTGCTCTGT	1				
P3_3	>hsa-mir-1303	GGGTCTTGCTCTGTTGCCAGGC	1				
P3_3	>hsa-mir-1303	TAGAGACGGGGTCTTGCTCTGT	1	ACGGGGTCTTGCTCTGTTGCCA	1		

## Data Availability

The pre-miRNAs data used to support the findings of this study have been deposited in the miRBase (release V20). The data are available from ftp://mirbase.org/pub/mirbase/20/hairpin.fa.zip. SNPs data of individual samples in 1000 Genome Project including 23 Chromosome data used to support the findings of this study have been deposited in the 1000 Genomes Project. The data are available from ftp://ftp.1000genomes.ebi.ac.uk/vol1/ftp/release/20130502/. The human GRCh37 reference sequence data (human_g1k_v37.dict, human_g1k_v37.fasta, and human_g1k_v37.fasta.fai) used to support the findings of this study have been deposited in ftp://ftp.1000genomes.ebi.ac.uk/vol1/ftp/technical/reference/. The miRNA sequencing data of individual sample in 1000 Genome Project used to support the findings of this study have been deposited in https://www.ebi.ac.uk/arrayexpress/experiments/E-GEUV-2/samples/.

## Supplementary Materials

Supplementary Materials There are three supplement data as follows: The 96 SNPs positions mapping to 90 pre-miRNAs of HG00096 is shown in Supplement Table 1. The SNP-pre-miRNA sequences of HG00096 can be shown as Supplement Table 2. The identification mature miRNAs of SNP-pre-miRNAs of HG00096 are shown as Supplement Table 3.

## References

[1] Krol J., Loedige I., Filipowicz W. (2010). The widespread regulation of microRNA biogenesis, function and decay. *Nature Reviews Genetics*.

[2] Ha M., Kim V. N. (2014). Regulation of microRNA biogenesis. *Nature Reviews Molecular Cell Biology*.

[3] Siomi H., Siomi M. C. (2010). Posttranscriptional Regulation of MicroRNA Biogenesis in Animals. *Molecular Cell*.

[4] Cammaerts S., Strazisar M., Dierckx J., Del Favero J., De Rijk P. (2016). miRVaS: a tool to predict the impact of genetic variants on miRNAs. *Nucleic Acids Research*.

[5] Sun G., Yan J., Noltner K. (2009). SNPs in human miRNA genes affect biogenesis and function. *RNA*.

[6] Hogg D. R., Harries L. (2014). Human genetic variation and its effect on miRNA biogenesis, activity and function. *Biochemical Society Transactions*.

[7] Muller H., Marzi M. J., Nicassio F. (2014). IsomiRage: From functional classification to differential expression of miRNA isoforms. *Frontiers in Bioengineering and Biotechnology*.

[8] Neilsen C. T., Goodall G. J., Bracken C. P. (2012). IsomiRs—the overlooked repertoire in the dynamic microRNAome. *Trends in Genetics*.

[9] Shukla V., Varghese V. K., Kabekkodu S. P., Mallya S., Satyamoorthy K. (2017). A compilation of Web-based research tools for miRNA analysis. *Briefings in Functional Genomics*.

[10] Guo L., Chen F. (2014). A challenge for miRNA: Multiple isomiRs in miRNAomics. *Gene*.

[11] Liu C., Zhang F., Li T. (2012). MirSNP, a database of polymorphisms altering miRNA target sites, identifies miRNA-related SNPs in GWAS SNPs and eQTLs. *BMC Genomics*.

[12] Bhattacharya A., Ziebarth J. D., Cui Y. (2014). PolymiRTS Database 3.0: Linking polymorphisms in microRNAs and their target sites with human diseases and biological pathways. *Nucleic Acids Research*.

[13] Maxwell E. K., Campbell J. D., Spira A., Baxevanis A. D. (2015). SubmiRine: assessing variants in microRNA targets using clinical genomic data sets. *Nucleic Acids Research*.

[14] Barenboim M., Zoltick B. J., Guo Y., Weinberger D. R. (2010). MicroSNiPer: a web tool for prediction of SNP effects on putative microRNA targets. *Human Mutation*.

[15] Thomas L. F., Saito T., Sætrom P. (2011). Inferring causative variants in microRNA target sites. *Nucleic Acids Research*.

[16] Deveci M., Çatalyürek Ü. V., Toland A. E. (2014). mrSNP: Software to detect SNP effects on microRNA binding. *BMC Bioinformatics*.

[17] Sablok G., Milev I., Minkov G. (2013). isomiRex: Web-based identification of microRNAs, isomiR variations and differential expression using next-generation sequencing datasets. *FEBS Letters*.

[18] Guo L., Yu J., Liang T., Zou Q. (2016). miR-isomiRExp: a web-server for the analysis of expression of miRNA at the miRNA/isomiR levels. *Scientific Reports*.

[19] Zhang Y., Zang Q., Xu B. (2016). IsomiR Bank: a research resource for tracking IsomiRs. *Bioinformatics*.

[20] Gong J., Liu C., Liu W. (2015). An update of miRNASNP database for better SNP selection by GWAS data, miRNA expression and online tools. *Database*.

[21] Bhartiya D., Laddha S. V., Mukhopadhyay A., Scaria V. (2011). miRvar: A comprehensive database for genomic variations in microRNAs. *Human Mutation*.

[22] Xiong H. Y., Alipanahi B., Lee L. J. (2015). The human splicing code reveals new insights into the genetic determinants of disease. *Science*.

[23] Leclercq M., Diallo A. B., Blanchette M. (2013). Computational prediction of the localization of microRNAs within their pre-miRNA. *Nucleic Acids Research*.

[24] Liu M. J., Wu S. H., Chen H. M., Wu S. H. (2012). Widespread translational control contributes to the regulation of Arabidopsis photomorphogenesis. *Molecular Systems Biology*.

[25] Gkirtzou K., Tsamardinos I., Tsakalides P., Poirazi P. (2010). MatureBayes: a probabilistic algorithm for identifying the mature miRNA within novel precursors. *PLoS ONE*.

[26] Guan D.-G., Liao J.-Y., Qu Z.-H., Zhang Y., Qu L.-H. (2011). mirExplorer: detecting microRNAs from genome and next generation sequencing data using the AdaBoost method with transition probability matrix and combined features. *RNA Biology*.

[27] Xuan P., Guo M., Huang Y., Li W., Huang Y. (2011). Maturepred: efficient identification of microRNAs within novel plant pre-miRNAs. *PLoS ONE*.

[28] Wu Y., Wei B., Liu H., Li T., Rayner S. (2011). MiRPara: a SVM-based software tool for prediction of most probable microRNA coding regions in genome scale sequences. *BMC Bioinformatics*.

[29] Terai G., Okida H., Asai K., Mituyama T. (2012). Prediction of conserved precursors of miRNAs and their mature forms by integrating position-specific structural features. *PLoS ONE*.

[30] Berezikov E. (2011). Evolution of microRNA diversity and regulation in animals. *Nature Reviews Genetics*.

[31] Karathanasis N., Tsamardinos I., Poirazi P., Patnaik S. (2015). MiRduplexSVM: a high-performing mirna-duplex prediction and evaluation methodology. *PLoS ONE*.

[32] Wang Y., Li X., Tao B. (2016). Improving classification of mature microRNA by solving class imbalance problem. *Scientific Reports*.

[33] Li J., Wang Y., Wang L. (2015). MatPred: computational identification of mature micrornas within novel pre-MicroRNAs. *BioMed Research International*.

[34] Genomes Project C., Auton A., Brooks L. D. (2015). A global reference for human genetic variation. *Nature*.

